# Mediastinal infections: diagnostic and therapeutic advances from traditional surgery to novel minimally invasive techniques

**DOI:** 10.3389/fmed.2025.1653443

**Published:** 2025-09-10

**Authors:** Yansong Xu, Guanbiao Liang, Chanyu Huang, Yuewu Wang, Zheng Liang, Yun Jiang, Cuiqing Huang, Junting Liu

**Affiliations:** ^1^Emergency Surgery Section, First Affiliated Hospital of Guangxi Medical University, Nanning, China; ^2^Thoracic Surgery Section, First Affiliated Hospital of Guangxi Medical University, Nanning, China

**Keywords:** mediastinal infections, diagnostic challenges, therapeutic challenges, mortality, precision medicine

## Abstract

Mediastinal infections present significant diagnostic and therapeutic challenges, contributing to highly variable mortality. Diagnostic dilemmas arise from complex anatomy and radiographic similarities to malignancies. Endobronchial Ultrasound-guided Transbronchial Needle Aspiration (EBUS-TBNA) and cultures are constrained by small samples, architectural distortion, low sensitivity, and slow results in special circumstances. Therapeutic obstacles include antibiotic resistance, poor antimicrobial penetration due to altered vascularity, and high surgical morbidity. Endobronchial ultrasound-guided transbronchial mediastinal cryobiopsy (EBUS-TMC) provides larger histologically preserved specimens; metagenomic next-generation sequencing (mNGS) achieves rapid sensitive pathogen detection; advanced imaging (Dual Energy Computed Tomography, DECT; Positron Emission Tomography/Computed Tomography, PET/CT) enhances lesion differentiation and intervention planning; while minimally invasive drainage, nanocarrier-based targeted antimicrobial delivery, and reconstructive techniques collectively reduce complications and improve therapeutic efficacy. Multidisciplinary integration of these innovations is advancing precision medicine approaches.

## 1 Introduction

Mediastinal infections represent a challenging category of clinical disease, posing significant difficulties throughout the diagnostic and therapeutic process with 1.8–33.1% mortality (1, 2). Diagnostically, the complex anatomy of the mediastinum complicates lesion localization, while the frequent radiographic overlap between different pathologies—such as infectious mediastinitis and neoplastic lesions—creates substantial diagnostic dilemmas (3). Although traditional diagnostic methods like EBUS-TBNA offer some value, their utility is limited by often yielding insufficient sample volume and compromised tissue architecture, which impacts pathological diagnostic accuracy (4). Microbiological diagnosis faces similar hurdles, particularly for deep-seated fungal infections like invasive pulmonary aspergillosis, where conventional cultures suffer from low positivity rates and prolonged turnaround times (4).

The spectrum of mediastinal infections is diverse, encompassing primary or secondary lymphoproliferative diseases, postoperative infections (bacterial/fungal), inflammatory diseases, and more (5, 6). The pathogens are primarily fungi (especially Candida species) and bacteria (gram-positive/gram-negative bacteria), with viruses and specific pathogens (such as Pneumocystis) being less common (6–8). The 2017 expert consensus of the European Association for Cardio-Thoracic Surgery points out that the key to the treatment of mediastinitis lies in the early administration of broad-spectrum antibiotics and promptly correcting drug use based on drug sensitivity (9). Therapeutic management encounters three major obstacles: First, antibiotic options are increasingly constrained by the emergence of drug-resistant pathogens (e.g., methicillin-resistant *Staphylococcus aureus*), rendering traditional regimens ineffective. Regional variations in antibiotic resistance pose significant challenges to the treatment of mediastinal infections (10, 11). Second, compromised antibiotic penetration into the mediastinum, attributed to its sparse vascularity and adipose-rich composition, frequently leads to therapeutically insufficient drug levels at infected foci—particularly in post-sternotomy cases with devascularized tissues. Finally, surgical intervention carries significant risks, as patients with post-sternotomy mediastinitis often present with tissue necrosis, and debridement procedures can further compromise chest wall stability (7). Collectively, these factors contribute to the persistently high mortality rates associated with mediastinal infections.

Recent years have witnessed the emergence of several innovative diagnostic and therapeutic strategies. In diagnostics, EBUS-TMC has significantly improved the diagnosis rate of rare mediastinal tumors like lymphoma (achieving diagnostic rates up to 89.8%) by procuring larger, more architecturally preserved tissue sample (3). Nanomaterial technology offers new possibilities for non-invasive diagnosis, with functionalized nanoparticles enabling highly sensitive detection of biomarkers within the infectious microenvironment (12). Targeted drug delivery systems, such as folate receptor-modified nanoparticles, demonstrate the ability to overcome the blood-tissue barrier, enhancing antibiotic concentration at the infection focus. Additionally, multidisciplinary team (MDT) approaches, integrating molecular diagnostics, image-guided navigation, and personalized pharmacotherapy, are transforming the landscape from traditional empirical treatment toward more precise management (13).

## 2 Limitations of traditional diagnostic and therapeutic approaches

The management of mediastinal infection faces multiple challenges: 1, Traditional microbial culture methods exhibit insufficient sensitivity, with blood or pus cultures often yielding false-negative results (14), making detection of rare or fastidious microorganisms difficult (15); 2, Computed tomography (CT) features of mediastinal abscesses overlap with those of malignancies (16), and invasive biopsy remains the gold standard for evaluating lymph node infection (17); 3, Conventional surgical debridement carries high risks for patients with comorbidities, while systemic antibiotics demonstrate poor local penetration (18). Leonardi et al. reported that 71% of descending mediastinitis cases require combined cervicothoracic incision, associated with high complication rates (19); 4, Culture of nontuberculous mycobacteria requires several weeks (20), and potassium hydroxide smear microscopy plus culture for fungal infections also suffer from low sensitivity and prolonged turnaround times (21); 5、Fungal mediastinitis in immunocompetent individuals may be overlooked due to atypical presentations (15), and conventional investigations often fail to trace the origin of hematogenously disseminated infections (22).

## 3 Advances in mediastinal abscess diagnosis

### 3.1 Pivotal role of CT in diagnosing mediastinal abscesses

Contrast-enhanced CT specifically delineates the enhancement pattern of the abscess wall and identifies potential complications, such as aortic aneurysm. Additionally, CT-guided interventions, potentially combined with endoscopic ultrasound (EUS), provide precise targeting of deep abscess cavities. This significantly enhances the success rate of drainage procedures and paves the way for subsequent surgical intervention (23). In terms of differential diagnosis, Multidetector Computed Tomography (MDCT) leverages its volumetric anatomical data to effectively distinguish abscesses from other space-occupying lesions, such as teratomas and **T**-lymphoblastic lymphoma, and to identify characteristic imaging signs (e.g., alterations in fat planes and gas shadows) (24). Furthermore, serial CT follow-up is paramount for therapeutic monitoring, as it enables both dynamic assessment of abscess size evolution and treatment response (25), and early detection of abscess formation resulting from the spread of deep neck infections into the mediastinum, thereby providing timely warning of recurrence or complications (25).

### 3.2 Emerging imaging biomarkers

Emerging imaging biomarkers refer to novel biological markers extracted through advanced imaging techniques (such as AI-based radiomics, molecular imaging, and functional imaging), primarily used for early disease diagnosis and disease progression monitoring. In the realm of imaging, DECT utilizes material decomposition algorithms to generate quantitative biomarkers, enabling noninvasive discrimination between benign and malignant anterior mediastinal masses (26). Furthermore, 18F-FDG PET/CT aids in differential diagnosis by assessing inflammatory metabolic activity; however, caution is warranted regarding potential false positives due to postoperative inflammation, and definitive diagnosis still requires histological confirmation (27, 28). For surgical planning, advanced MRI techniques, such as cine-MRI for evaluating cardiac dynamic involvement and high-resolution T1 turbo spin-echo (T1 TSE) sequences, clearly delineate tumor infiltration into critical mediastinal structures, thereby optimizing surgical decision-making (29, 66). Despite the proven utility of imaging biomarkers in diagnosing mediastinal infections, the field currently lacks a closed-loop pathway converting technical outputs into clinical decisions, with no established framework for translating biomarker data to therapeutic actions.

### 3.3 The groundbreaking value and challenges of mNGS in pathogen diagnosis of mediastinal infections

mNGS significantly enhances the diagnostic efficacy for pathogens in mediastinitis by virtue of its culture-independent nature. Crucially, it enables precise identification of potential pathogens in culture-negative cases, providing critical evidence for targeted antibiotic selection and improved prognosis. Compared to traditional bacterial culture, this novel microbiological diagnostic technology offers multifaceted advantages (30). 1, mNGS overcomes the limitation of high false-negative rates associated with conventional culture, successfully detecting pathogens in culture-negative mediastinitis and significantly increasing the detection rate, with a reported sensitivity of 79.5% (31). 2, It bypasses the need for culture, thereby enabling the direct detection of fastidious or difficult-to-culture pathogens. 3, mNGS dramatically shortens the diagnostic time compared to conventional culture (days/weeks) and even techniques like qPCR, reducing the detection cycle to mere hours (32, 33). 4, mNGS provides unbiased, pan-pathogen screening, simultaneously detecting bacteria, viruses, fungi, and parasites (34). While mNGS offers diagnostic benefits for infectious diseases, its implementation in routine mediastinal infection management may be constrained by substantial sequencing costs (29).

### 3.4 Interventional diagnostics: precision sampling beyond non-invasive imaging

EBUS-TBNA, as a minimally invasive technique, plays a critical role in diagnosing mediastinal infections. Compared to traditional mediastinoscopy, EBUS-TBNA offers significant advantages, including reduced invasiveness and fewer complications (35). However, while EBUS-TBNA demonstrates high diagnostic accuracy for malignancies, its utility in infectious diseases is limited. These limitations primarily stem from the small tissue sample volume obtained, which may compromise microbial culture yield, and the potential risk of rare postoperative mediastinal infections (e.g., mediastinal abscess due to bronchial wall microperforation) (35, 36). To enhance its diagnostic value in mediastinal infections, researchers have developed modified techniques. EBUS-TBNA demonstrates superior diagnostic yield for mediastinal malignancies such as lymphoma (sensitivity >89%), followed by infection diseases (9.9%) (37). However, in infectious mediastinal pathologies (e.g., granulomatous infections), current evidence remains limited to small retrospective series. Its use should therefore be considered investigational, reserved for cases where conventional microbiological sampling fails to establish a diagnosis (37). Research has found that in addition to its high safety profile, EBUS-TMC achieves a diagnostic accuracy rate of 89.59% (38), and EBUS-guided transbronchial forceps biopsy as a complementary method for increased tissue yield. Beyond diagnosis, EBUS-TBNA holds therapeutic potential for drainage (39). EBUS-TMC demonstrates superior diagnostic performance for rare mediastinal diseases. However, its procedure requires adjunctive cryobiopsy techniques, demands greater operator expertise, and relies on specialized equipment, potentially limiting its adoption in resource-limited settings (3, 40).

## 4 Limitations of traditional interventions

### 4.1 Challenges of antibiotic therapy in mediastinal infections

The effective management of mediastinal infections with antibiotics faces several significant challenges. Firstly, accurately identifying the causative pathogens is difficult due to the deep-seated location and complex microbial ecology of the mediastinum, often necessitating invasive sampling. Moreover, achieving adequate antibiotic penetration into infected mediastinal tissues and abscesses is frequently suboptimal, leading to insufficient drug concentrations at the infection site (41). Research showed the concentration of aminoglycosides in mediastinal abscess is only 18% of serum (42). Furthermore, the increasing prevalence of multidrug-resistant organisms poses a substantial therapeutic hurdle (43). Additionally, the formation of biofilms on infected tissues or foreign materials significantly reduces antibiotic efficacy. Finally, determining the optimal duration of therapy remains controversial, as prolonged courses carry risks of toxicity and antimicrobial resistance, while insufficient duration risks treatment failure; standardized regimens are lacking. Therefore, successful antibiotic therapy for mediastinal infections requires a multifaceted approach. This includes meticulous microbiological diagnosis guided by culture and susceptibility testing (when feasible), careful selection of agents with optimal penetration profiles (potentially at higher doses), consideration of combination therapy for resistant pathogens or biofilms, individualized treatment duration based on clinical response and infection type, and close monitoring for efficacy and adverse effects.

### 4.2 Indications and limitations of surgical debridement

Radical debridement and drainage constitute the cornerstone of management for deep mediastinal infections, including mediastinitis, particularly when microbiologically confirmed by culture or gross intraoperative evidence of infection (7). In fungal mediastinitis post-cardiac surgery (e.g., *Candida/Aspergillus* spp.), adjunctive surgical intervention alongside systemic antifungals is essential—despite mortality rates approaching 60% in immunocompromised cohorts, with survival favoring younger patients (<50 years), those with lower BMI (<25), and non-septic presentations (6). Similarly, bacterial mediastinitis (notably *Staphylococcus aureus*) carries a grave prognosis, exacerbated by methicillin resistance (9). For infections without septicemia, encapsulated foci warrant surgical debridement if persistent after 48 h of appropriate antibiotics (e.g., <50% CRP decline) (9). Conversely, conservative management remains first-line for: (1) superficial sternal wounds, (2) non-necrotizing mediastinal lymphadenitis, and (3) chronic fibrosing mediastinitis, barring disease progression.

Anterior mediastinal infections invading critical structures (e.g., right ventricle, coronary vasculature) often preclude radical resection due to inseparable adherence, necessitating image-guided drainage and targeted therapy. Concurrently, sternal osteomyelitis with mediastinal extension faces technical constraints, where negative-pressure wound therapy bridges staged reconstruction. VAC therapy dominates conventional drainage, providing superior clinical outcomes: 39% reduction in reoperation needs, 34% shorter ICU stays and 62% lower readmission rates for recurrent infections. In prior mediastinal radiotherapy recipients—where tissue friability and impaired healing amplify surgical risks—endoscopic approaches may offer superior safety over open procedures (44).

## 5 Advances in minimally invasive management of mediastinitis

### 5.1 Bronchoscopic therapeutic techniques

Bronchoscopy enables transbronchial access to mediastinal lesions via EBUS-guided needle aspiration or transparenchymal navigation, providing samples for pathology and microbiology. Furthermore bronchoscopy facilitates targeted local drug delivery (e.g., vasoconstrictors, antifibrinolytics) to control bleeding or infection (45), and the local instillation of antifungal agents (such as amphotericin B) for refractory mediastinal infections (46). Despite its minimally invasive advantages, several important considerations apply. Firstly, international guidelines have not yet established consensus regarding its application in severe respiratory infections. Secondly, MDT collaboration is essential for appropriate patient selection and indication assessment. Finally, a high index of suspicion remains crucial for Aspergillus-related mediastinal abscesses, even in immunocompetent patients. While valuable for diagnosis and some specific therapeutic applications within the airways, its role as a primary therapeutic tool for deep mediastinal infections remains unproven and should be considered highly investigational outside specific, carefully documented cases.

### 5.2 Application of thoracoscopic drainage

Video-assisted thoracoscopic surgery (VATS) for mediastinal procedures offers advantages of relative simplicity and minimal invasiveness. Compared with traditional surgery, VATS is associated with significantly reduced intraoperative blood loss (*p* < 0.001), fewer postoperative complications (*p* = 0.048), and shorter durations of chest tube drainage and hospital stay (p < 0.001) (47). Additionally, VATS drainage reduces the risk of accidental chest tube displacement (3.9% vs. 10.1%), thereby lowering the risk of treatment failure (48). The key technical advantage of VATS lies in its ability to achieve adequate debridement and drainage of the mediastinum and pleural cavity through small incisions, making it particularly valuable for deep-seated infections or complex cases complicated by empyema. However, important limitations still exist. VATS can be restricted by poor visualization and limited working space in cases of extensive adhesions or major vascular involvement within complex mediastinal infections. Therefore, prudent selection of VATS or consideration of hybrid procedures (combining VATS with limited open access) is recommended for such challenging scenarios.

While VATS is undoubtedly a valuable tool, its application in the complex mediastinum requires careful patient selection by experienced surgeons, with a clear understanding that its benefits are maximized in suitable cases and may be significantly reduced or negated in the very scenarios (extensive adhesions, major vascular involvement) where deep infections often present.

### 5.3 Application of the sternocleidomastoid (SCM) flap in mediastinal infection management

The SCM flap is a valuable option for reconstruction following extensive mediastinal surgery due to its anatomical proximity to the mediastinum and relative technical ease of harvest. Evidence supports that flap reconstruction (including the SCM flap) significantly reduces infection rates post-mediastinal surgery. For instance, a study involving high-risk patients demonstrated a statistically significant reduction in infection rates (*p* < 0.05) in the flap reconstruction group compared to the non-flap group, despite a higher proportion of patients in the flap group having undergone radiotherapy (49). Moreover, the SCM flap, particularly when combined with negative pressure wound therapy, has proven effective as a bridging therapy in the management of deep sternal wound infections (50). While historical concerns existed regarding the vascular reliability of the SCM flap, its efficacy in mediastinal reconstruction is now well-established. However, for complex deep infections involving large dead spaces, the SCM flap often requires supplementation with larger flaps such as the omental or pectoralis major flap for adequate coverage. Should flap necrosis occur, salvage reconstruction using free tissue transfer is a viable option. While the SCM flap is indeed a valuable tool in the reconstructive armamentarium, its presentation here leans toward over-optimism by underplaying its drawbacks and over-interpreting the cited evidence concerning its specific contribution to infection reduction.

### 5.4 Local antifungal irrigation

Local irrigation following surgical debridement offers a potential solution by removing necrotic tissue and enhancing drug penetration, particularly in poorly vascularized abscess regions (51). Evidence suggests specific antifungal combinations may be beneficial: Liposomal amphotericin B combined with flucytosine is the preferred regimen for central nervous system infections and may also be applicable to cases with mediastinal extension (52). Furthermore, triple-drug therapy (amphotericin B + posaconazole + flucytosine) has demonstrated significantly improved survival in Cladophialophora bantiana infections, highlighting the value of combination regimens for refractory mediastinal infections (52). Therefore, local antifungal irrigation holds promise as an adjunctive therapy to surgical intervention and systemic treatment in mediastinal infections. However, its clinical application necessitates further investigation to validate optimal protocols (including agent selection, irrigation concentration/frequency) and synergistic effects with systemic antifungals (53). Local irrigation (e.g., voriconazole nanomicelles) is suitable for superficial infections, but exhibits limited penetration into deep-seated mediastinal infections (such as fungal mediastinitis post-cardiac surgery), often necessitating adjunctive systemic therapy (54). However, it significantly overreaches by implying promise based on evidence that is either from different anatomical sites (CNS) or for specific rare pathogens (C. bantiana), which does not directly support efficacy in the mediastinum for common fungi. The critical limitations regarding penetration depth, lack of protocol standardization, unproven synergy, potential risks, and the lack of direct clinical evidence for mediastinal irrigation efficacy are substantial.

### 5.5 Nanocarrier-based targeted drug delivery

Nanocarriers offer a promising strategy for targeted drug delivery in mediastinal infections by penetrating complex tissue barriers (such as fibrotic or inflamed mediastinal regions) to transport therapeutic agents directly to deep-seated infectious foci (55). Crucially, specific nanocarrier designs demonstrate potent antimicrobial effects. Preclinical study demonstrated that inhalable chitosan-fusogenic nanocarriers (CFusoN) achieve 99.9% bactericidal efficiency *in vitro* against biofilm-embedded MRSA (56). Targeted delivery via nanocarriers enables reduced antibiotic dosing and mitigates resistance risks (57). Furthermore, inhalable nanocarriers (e.g., liposomes, polymeric nanoparticles) enable the direct delivery of drugs to the mediastinum via the respiratory tract, significantly reducing the risk of systemic toxicity (58). However, despite their considerable potential in managing mediastinal infections, key challenges must be addressed before widespread clinical adoption. These include mitigating the impact of the protein corona effect, optimizing scale-up manufacturing processes, and facilitating successful clinical translation. The long-term safety profile of amphotericin B (AmB) nanocarriers warrants further validation, while susceptibility of certain pathogens (e.g., Candida spp.) to lipid-based formulations remains suboptimal (59, 60). Although CFusoN has a 99.9% bactericidal rate against MRSA biofilm *in vitro*, *in vivo* mediastinal infection models show that mediastinal fibrotic tissue reduces nanoparticle diffusion efficiency, resulting in a clinical remission rate of only 48%. In addition, nanocarriers may accelerate drug resistance evolution.

## 6 Future research progress

### 6.1 Potential of artificial intelligence (AI)-assisted diagnosis in mediastinal infections

AI enhances diagnostic efficiency while rigorously preserving patient privacy (61). Notably, these systems utilize deep learning models to achieve precise segmentation and classification of mediastinal lesions, demonstrating strong generalizability in multi-center validation studies. A retrospective, sequential, multireader, multicase study indicates that AI assistance significantly improves diagnostic quality by increasing the sensitivity of mediastinal abnormality detection from 84.3 to 90.8% (62). Artificial intelligence research has the advantages of high data standardization, clear commercial value, and strong technology compatibility in tumor diagnosis. On the contrary, the application of AI-assisted diagnosis in mediastinal infection is still lacking. The main causes may be fragmented cases with high heterogeneity of mediastinal infections, rapid dynamic changes, and reliance on the gold standard for invasive procedures.

Although AI-assisted diagnosis for mediastinal infections remains an understudied field, its technical foundation has begun to emerge: successful developments in dynamic imaging analysis models for pneumonia, and surgical infection monitoring tools have established a robust methodological framework for dedicated AI systems targeting mediastinal infections (63). Future breakthroughs should prioritize dynamic pathological modeling as the core focus, supported by multicenter data ecosystem development, ultimately enabling the transition from reactive diagnosis to proactive intervention.

### 6.2 Advancements in molecular diagnostic technologies

CRISPR-Cas systems represent the next generation of molecular diagnostics. Their advantages, including high specificity, isothermal operation, absolute quantitation, rapid detection, and applicability to universal DNA/RNA targets, position them as transformative platforms (64). Furthermore, overcoming diagnostic challenges posed by microbiome interference requires integrating pathogen detection with analysis of the host immune response (e.g., transcriptomic profiling, immune function assays). Portable integrated platforms, exemplified by “lab-on-a-disk” magnetic digital microfluidics, enable fully automated sample-to-answer processing, making them suitable for point-of-care or emergency settings (65). Addressing false negatives arising from genomic variations (e.g., mutations in primer-targeted regions) necessitates developing detection methods independent of primer conservation and utilizing whole-genome sequencing to monitor escape variants. Finally, the convergence of emerging technologies – such as lipidomics, transcriptome analysis, and infrared molecular fingerprinting – holds promise for discovering novel diagnostic biomarkers and advancing personalized diagnostics.

## 7 Conclusion

Mediastinal infections carry a high mortality rate, and their diagnosis and treatment face three major challenges: difficulty in pathogen diagnosis; prominent therapeutic bottlenecks; limitations of conventional techniques. Current breakthroughs lie in: (1) mNGS technology significantly improving pathogen detection rate and timeliness; (2) Innovations in minimally invasive techniques; (3) Targeted drug delivery systems. The future requires integrating predictive AI modeling and CRISPR-based molecular diagnostics to enable precision interventions.
